# From body to world: empathy and the transformative power of cinematic imagination

**DOI:** 10.3389/fnhum.2024.1363720

**Published:** 2024-03-13

**Authors:** Isabel Jaén Portillo

**Affiliations:** Department of World Languages and Literatures, Portland State University, Portland, OR, United States

**Keywords:** empathy, prosocial behavior, empathy-altruism hypothesis, embodied simulation, emotions, enactment, film and embodiment, cinema/film

## Introduction

The transformative power of fiction has been acknowledged since antiquity (Jaén and Simon, 2012) and technologies such as cinematography have amplified it. This power resides in its capacity to move us, to change our emotional states. Moreover, the audiovisual and narrative strategies employed in film fictions allow us to experience emotions not only intensely but also safely. Our participatory responses are both elicited and modulated by the fictional “crafted” quality of the stories that we witness on the screen. What happens before our eyes does not pose a threat to us and does not need our intervention. Yet, it seems quite real to our brains and may transform us, by allowing us to adopt different cognitive-affective perspectives, which may result in prosocial behaviors, that is, actions oriented toward the benefit of others.

## The neurocognitive study of film

Research on our responses to moving images has been concerned with what happens inside of our body and how these phenomena correlate with other bodies, measuring for example inter-subject correlation or ISC (Hasson et al., 2008) and employing neuroimaging techniques (e.g., fMRI and EEG), which may be combined with other methods such as self-reports. Emerging techniques such as mobile EEG allow for improved conditions of study (e.g., subjects being able to watch a film in a more natural setting as opposed to lying still in an fMRI machine) and hold promise to deepen our understanding of the neural underpinnings of film. These studies need to be considered along with the exploration of the contextual aspects of our engagement with films beyond what happens in our embodied minds, not simply in relation to other minds but also to the wider social and cultural human networks, artifacts, and circumstances that they interact with and are part of.

Other studies on film and cognition carried out at the crossroads of the humanities and the sciences (e.g., Plantinga and Smith, 1999; Oatley, 2013; Tan, 2013; Gallese and Guerra, 2020) have focused on the emotional aspects of the cinematic experience: how movies engage our emotions and what are the implications of the emotional journey we undergo as audiences, including transformative effects within sociohistorical contexts (Jaén, 2017, 2018). While safely feeling when immersed in movies is regarded as a pleasurable experience, film fictions are more than just a thrill of myriad emotions for our momentary entertainment: they are powerful stories engineered by filmmakers to make us not only feel but also reflect. Through film, we might be able to adopt a new perspective regarding an urgent, controversial, or problematic issue, we might change our minds, and we might even attempt to change others' or engage in prosocial actions of different kinds (e.g., volunteering for a cause, donating money through an organization). In this sense, film fictions may provide us with a moral education that is mediated by our emotional engagement with the characters, as other types of fiction do (see Hakemulder, 2000; on moral education, see Camassa, 2024). Arguably, film fictions are particularly effective at eliciting emotions resulting in moral transformation, since they tap directly into our prelinguistic abilities to mimic and understand others through their faces and bodies, that is, to empathize with them.

## Empathy and the cinematic imagination

Indeed, in exploring how we engage in filmic fictions, how moving images impact us, we must first consider that the cinematic imagination is essentially empathic, that is, our capacity to feel with others is at the core of the experience with filmic artifacts, just as our everyday cognitive activity in our social environments is. In fact, our ability to follow the actions and emotions of characters on a screen relies on the same neural circuits and processes that make possible our real real-life interactions (see Gallese and Guerra, 2020). In this regard, 4E approaches to cognition, highlighting its embodied, embedded, enactive, and extended nature—and 5E approaches, including emotion as the fifth “E” (Troncoso et al., 2023)—help us not only to explore the multidimensionality of embodied empathy but also understand our condition of spectators as dynamic entities immersed in a surrounding world. Furthermore, they allow us to frame the interdisciplinary study of human cognition in relation to cultural phenomena such as literature and film (see Mancing and William, 2022).

But what are we talking about when we talk about empathy in the context of film studies? First, we must acknowledge that empathy is a complex and multifaceted phenomenon (Batson, 2009), for which several definitions and explanatory models have been proposed from diverse disciplines. A definition that captures the complexity of the notion of empathy, including not only its cognitive-emotional aspects but also its social-behavioral ones, and, thus, relates closely to our discussion is “the natural capacity to share, understand, and respond with care to the affective states of others” (Decety, 2012, p. 7). How do we explore and discuss these different aspects of empathy (sharing, understanding, and responding with care) in relation to the experiencing of films? Below I introduce briefly three models or approaches, including my own, that have been proposed to account for cinematic empathy and that are particularly helpful to frame current and future conversations about our engagement with film and its transformative potential.

## Discussion: three models/approaches for the study of cinematic empathy

(1) Embodied simulation (Gallese and Guerra, 2020): according to this model, “our visual experience of the world is the outcome of multimodal integration processes in which the motor system is a key player” (Gallese and Guerra, 2020, p. 20). Hence, our aesthetic experience of images and our understanding of the actions and intentions of characters are rooted in our somatosensory system. Mirror mechanisms trigger an embodied simulation of what we are observing on the screen, where the facial expressions of characters, available and enhanced through close-ups, are key to the observer's bodily response, engaging facial muscles as well as multiple brain areas related to motor and emotional processes. This “liberated simulation” (we simulate but do not perform the actions we observe, since our motor systems are not activated completely, nor with the same intensity) is at the core of our pleasurable (and safe) engagement with cinematic fictions. It is important to note that “our brain-body responses are modulated by cultural, historical, contextual, and idiosyncratic factors at both the psychological and physiological levels” (Gallese and Guerra, 2020, p. 89). While the main contribution of the embodied simulation approach is to make us aware of the somatosensory origins of the changes we undergo when we empathize with others, its consideration of context, as well as its claim to proceed from neural circuits to intersubjectivity, aligns it with phenomenological approaches to human cognition, going beyond body-focused approaches, and paving the way for a comprehensive view of cinematic empathy.
(2) Resonance-enactment (Tan, 2013): this model relates to two main perspectives on Theory of Mind, our ability to “read” and make sense of the mental states of others, their beliefs and intentions in order to navigate our social worlds: Simulation Theory (see Gordon, 1986)—we put ourselves in the others' shoes, we simulate being them—and Theory Theory (see Gopnik and Meltzoff, 1997)—we use our knowledge of how minds work to infer their mental states, we theorize about them. Tan's proposal is based on work by Goldman (2006), Decety (2007), and others. It seeks to integrate the automatic basic empathy processes that include emotional contagion and mimicry (resonance) with higher order appraisal processes (willful intentional empathy) that require inferring, reasoning, imagination, and introspection (enactment). In considering this model, we must presume the existence of a Theory of Body (see Mancing, 2016) in addition to a Theory of Mind (see Zunshine, 2006) in the spectators' engagement with fictional worlds. When watching a film, we not only simulate the expressions and actions that we perceive but also “read” the bodies and minds on the screen, attempting to infer their thoughts, emotions, and intentions. The film narrative guides our enactment of the characters' inner lives, creating expectations about their goals, plans, emotional reactions, etc. An emphasis on the narrative aspects of enactment connects this approach to enactivist perspectives on empathy that stress the dynamics between basic empathy based on enactive perception—other-oriented—and higher order empathy based on narrative imagination—personal and cultural narratives are employed to understand others in their contexts—(see Gallagher, 2012; Gallagher and Gallagher, 2020). In this respect, the resonance-enactment model provides a useful frame to explore the role of plot in our empathic responses to film and how spectators navigate film fictions according to expectations created by certain genres.
(3) Cinema of empathy (Jaén, 2018)^1^: this model emphasizes the embodied nature of our cinematic immersion by drawing on notions such as the prominence of the human face in the “scene of empathy” (Plantinga, 1999; p. 239)—a film strategy intended to elicit empathic emotions in the spectator—and the transmission of affect via inter-body physiological processes of alignment of a person's or a group's nervous and hormonal systems (Brennan, 2004; p. 9). Mood (see Smith, 2003) and genre (films depicting human rights abuse where grouped bodies are prominently featured, forming a collective protagonist entity) are foregrounded as framing parameters in the empathic experience of these films. We may think of cinema of empathy as a form of filmmaking that is mainly “affect-driven,” where emotion—inner but also, and particularly, shared—occupies a central role, rather than plot and action. The plot tends to constitute, in fact, a background to the foregrounded affected states of the characters, and it is often based on a historical event that the audience may be familiar with (e.g., WWII). This “liberates” to some extent spectators from having to focus on events, allowing them to concentrate on the affective states of characters. Guided by the filmmaker's cinematic strategies, intended to make us care, we follow their emotional reactions to their circumstances and watch them develop emotionally, as they cope with their fates. In addition to all the contextual elements that filmmakers employ to guide our attention and emotions (music, lighting, movement, camera angle, editing, etc.), cinema of empathy strategies rely on closeups of facial expressions and the affective energy generated by the aligned bodies on the screen to provide emotional cues. These contribute to create a mood that frames and mediates the embodied empathic response of spectators, who simulate and enact the emotional states as they follow them on the screen. This sustained state of mind or “empathic” mood may facilitate moral reflection around an ethical-socio-political issue presented in the film (see also Plantinga, 2012). By focusing on films that tap into large scale human abuse and collective trauma and deploy strategies that may change audiences' minds and behaviors, the cinema of empathy model provides a frame to explore the potential transformative effects, as well as the limits, of empathic responses to films in a wider context. Ethical and ideological factors are considered both synchronically (in a given cultural context) and diachronically (from a historical perspective) at the individual and collective levels of consciousness. In doing so, it revisits the empathy-altruism hypothesis (Batson, 2012), bringing back to the table the question of whether fictional narratives can help us care for the problems of others and make our world a better place. Finally, it also considers narrative empathy models that stress problematic phenomena such as empathic inaccuracy, failed empathy, and false empathy (Keen, 2006, 2007). Indeed, since the potential empathic effect of filmic strategies depends on physiological, cultural, and sociohistorical factors in individuals and groups, a direct correlation between filmmakers' empathic intention, empathic filmic strategies, and empathic audience response cannot be established.

## Conclusion

Empathy is central to the study of our engagement with film fictions and the models described above provide a robust frame to explore our empathic responses to the stories we follow on a screen. Although we need more studies and perspectives to understand the cinematic imagination and its transformative power, these models help us provide the grounds for a comprehensive theory on film empathy that connects the embodied mind to its social, cultural, and historical contexts, shifting our focus from body to world.

## Author contributions

IJ: Writing—original draft, Writing—review & editing.

## Footnotes

^1^For an extended discussion of the Cinema of Empathy model, including examples from the historical memory film genre, see Jaén (2018).
